# Chagas disease in the United States: a call for increased investment and collaborative research

**DOI:** 10.1016/j.lana.2024.100768

**Published:** 2024-05-17

**Authors:** Nelson Iván Agudelo Higuita, Norman L. Beatty, Colin Forsyth, Andrés F. Henao-Martínez, Jennifer Manne-Goehler, US Chagas Research Consortium, Daniel Bourque, Natalie M. Bowman, Malwina Carrion, Christina Coyle, Madolyn Dauphinais, Kelly DeToy, Robert Gilman, Davidson H. Hamer, Jesica Herick, Salvador Hernandez, Claudia Herrera, Rachel Marcus, Sheba Meymandi, Melissa Nolan, Katherine Reifler, Adrienne Showler, Paula Stigler Granados, Anshule Takyar, Kawsar Talaat, Shilah Waters, Alyse Wheelock

**Affiliations:** a Department of Medicine, Section of Infectious Diseases, University of Oklahoma Health Sciences Center, Oklahoma City, OK, USA; b Instituto de Enfermedades Infecciosas y Parasitología Antonio Vidal, Tegucigalpa, Honduras; c Department of Medicine, Division of Infectious Diseases and Global Medicine, University of Florida College of Medicine, Gainesville, FL, USA; d Emerging Pathogens Institute, University of Florida, Gainesville, FL, USA; e Drugs for Neglected Diseases Initiative–North America, New York, NY, USA; f Division of Infectious Diseases, Department of Medicine, University of Colorado Anschutz Medical Campus, Aurora, CO, USA; g Division of Infectious Diseases, Brigham and Women’s Hospital, Harvard Medical School, Boston, MA, USA

**Keywords:** Neglected tropical diseases, Chagas disease, Trypanosoma cruzi

## Abstract

Chagas disease, caused by the protozoan *Trypanosoma cruzi*, is a highly overlooked parasitic infection within the United States. It affects an estimated 300,000 individuals, often remaining asymptomatic for years before triggering severe complications such as cardiomyopathy in 30-40% of cases. While many contract the disease in Latin America, its transmission by local vectors in the southern U.S. presents a significant challenge. Unfortunately, limited access to diagnosis and treatment persists, alongside unresolved gaps in healthcare systems and disease pathogenesis. In this viewpoint, we discuss the need for focused research and public health initiatives, with U.S. research institutions playing a crucial role in developing new treatments and identifying biomarkers. Furthermore, investigating the genetic variations of *T. cruzi* between North and South America is vital for improving diagnostic and treatment strategies. Urgent action is required to implement national and local programs, bolstering healthcare responses and advancing research efforts.Q4As per journal style section heading ‘Introduction’ is mandatory, hence we have introduced the heading. Please check, and correct if necessary.ResolvedQ5If there are any drug dosages in your article, please verify them and indicate that you have done so by initialing this query.ResolvedQ6Please supply the year of publication.ResolvedFootnoteView Edit Log9

## Introduction

Chagas disease is a chronic parasitic infection endemic to the Americas which affects almost 6 million people worldwide, including an estimated 326,000–347,000 in the United States.^1^^,^^2^ Whilst most US cases were acquired in Latin America, both vector and congenital transmission of the causative agent, *Trypanosoma cruzi,* have been documented in the US. An estimated 22–108 infants are born in the US annually with congenital Chagas while 10,000 people may have been infected by local triatomine insects.^3^ The acute phase is often asymptomatic or confused with other febrile illnesses, so most people are unaware they are infected. If untreated, Chagas disease can lead to heart failure and other complications which cause significant morbidity and mortality, and a heavy annual economic toll estimated at over $148 million in healthcare costs and 27,000 disability adjusted life years,^4^ more than any other parasitic infection in the Americas. Nonetheless, important questions about epidemiology, diagnosis, and clinical management remain unanswered, with few public health resources dedicated to awareness and education, control of the disease, and care of affected people. Currently, less than 1% of *T. cruzi*-infected people in the US access testing and treatment.^5^^,^^6^

Chagas disease often has a lengthy latent period before long-term clinical complications manifest. The indeterminate form of the chronic phase of the disease is asymptomatic, and reliable biomarkers of progression are still lacking. Some individuals can remain in this stage for decades, although endemic areas can observe annual rates of up to 2% of cardiomyopathy onset.^7^ Once cardiomyopathy is established —even minor— the overall yearly mortality could reach 8%,^8^ and etiological treatment may no longer be effective. The parasite has marked cardiac tropism, and heart tissue infection is universal during acute infection.^8^ Heart tissue PCR detects *T. cruzi* amastigotes in 33–85% of patients with chronic Chagas cardiomyopathy.^9^

## US institutions could play an important role in answering global research questions

Research institutions in the US have already forged key collaborations and played an important role in basic and clinical research, such as exploration of therapeutic vaccines.^10^^,^^11^ However, with increased support that role could be expanded toward addressing pressing questions around disease pathology, biomarkers, and drug therapy.

### Biomarkers

We lack biomarkers for reliably assessing cure or accurately predicting which people with *T. cruzi* infection will develop complications. Myocardial involvement during acute or chronic infection urges studying biomarkers for predicting cardiomyopathy risk and associated mortality. Additionally, identification of biomarkers that could predict risk of the relatively high rates of sudden death from arrhythmias in individuals without symptomatic heart disease could help develop life-saving preventive interventions. Increasing collaboration among endemic Latin-American research centres and US institutions can enhance the biorepository of Chagasic hearts and tissues to launch studies of RNA signature expressions as biomarkers. The discovery of valuable biomarkers of disease progression and of disease-associated mortality can aim at the development of safer or more effective drug treatment, screening, and vaccines.^12^ Furthermore, any actionable therapeutic gains and insights on Chagas cardiomyopathy could inform management of other infectious or non-infectious forms of cardiomyopathy.

### Drug development

The only drugs effective against *T. cruzi*, benznidazole and nifurtimox, are decades old and produce frequent side effects that cause one in five patients to abandon treatment. Despite having the sixth highest burden of disease worldwide, the US has not yet hosted international clinical trials for new treatments, although the NIH has supported trials in Latin America with involvement of US institutions.^13^ US academic institutions and biotechs can—and do–play a role in early stage research, but with few new antitrypanosomal drug candidates on the horizon, more resources and concerted involvement are needed to replenish the current pipeline.

### *T. cruzi* genetic diversity

*T. cruzi* is a genetically heterogeneous species. Seven evolutionary or discrete typing units (DTUs), termed TcI to TcVI and the more recently described TcBat, are currently recognised.^14^ Apart from the influence of the host’s genetics and immune response to the parasite, a possible correlation between the genotype of the infecting strain and its effect in the (1) susceptibility of the human host to different forms of transmission (e.g. oral, congenital), (2) determination of clinical phenotypes, and (3) response to treatment have been inferred but not conclusively demonstrated in different studies.^14^ Furthermore, the differing geographic distribution of the diverse genotypic strains of *T. cruzi* leads to variations in the human host’s humoral response, translating to regional discrepancies in the performance of serological testing.^15^ Preliminary data indicate that mixed infections with different DTUs in locally acquired human cases might not be uncommon. Further, there are crucial knowledge gaps regarding the pathology and potential drug resistance of *T. cruzi* strains in North America.^16^

## Research questions specific to North America and the US

### Climate change

Global warming is an important factor affecting the distribution and frequency of vector-borne diseases.^17^ In North America, a potential increase in Chagas disease risk is predicted and related to expansion of suitable habitats for the most common vectors.^18^^,^^19^ A changing climate could open up new ecological niches for some vector species, which may increase the risk of transmission to humans. Moreover, climate change especially impacts the same marginalized communities that bear a disproportionate burden of Chagas disease. As land use and migration patterns shift in response to climate change, new vector transmission scenarios may emerge.^18^^,^^19^ Sustainable investment in surveillance and control programs under the umbrella of a One Health approach through local health departments and education to improve public awareness are fundamental interventions for a changing climate. In the US, studies examining circulating DTUs in sylvatic habitats, domesticated animals, and in the human infected population are scarce. Further research is also needed to understand how climate change will impact parasite biology and the distribution of important host species in North America.

### Disease burden and transmission

A few studies have assessed the prevalence of Chagas disease in specific locations.^20^, ^21^, ^22^ However, given the lack of routine screening in healthcare, more epidemiological data are needed to better understand the disease burden. Models of care that can efficiently identify people with the infection and provide treatment options are another urgent need.

Although the estimated number of autochthonous *T. cruzi* infections is small (∼ 10,000 prevalent cases) in comparison to the number of cases in immigrants, CDC estimates that 5.5%–7.5% of confirmed infections in blood donors are related to locally acquired infection.^3^^,^^23^ The US is thus not a “non-endemic” country in the strictest sense; acknowledging this would be a powerful symbolic statement highlighting the impact of neglected diseases in high-income countries, but enhancing US leadership in the global struggle to control them.

The natural history of autochthonous Chagas disease in the US is poorly understood and might differ from that of Latin America. For example, reinfections may play a role in developing and exacerbating chronic Chagas cardiomyopathy, an event that is unlikely in most of North America when compared to highly endemic areas of Latin America.^24^ Longitudinal studies to identify other drivers of progression specific to the US population are needed.

### Diagnostics

New diagnostics represent another critical need. Diagnosis is challenging with only four tests having FDA clearance for clinical use, with varying availability in the market. Tests with strong performance in South American populations may not translate to North America. Recent studies have provided key insight,^22^^,^^25^ but there is a need for more information to understand variations in diagnostic performance in the heterogeneous US at-risk population.

## Strengthening research collaborations

The creation of a national consortium of clinics and biorepositories could enable the application of precision medicine through broadscale human and pathogen genome sequencing of a large number of specimens. The consortium could help circumvent many of the enduring challenges related to the suboptimal performance of diagnostics, understanding of the natural history of the disease, risk stratification, and early assessment of response to therapy. Development of new diagnostic modalities such as metagenomic next-generation sequencing or the combination of highly multiplexed target capture and next-generation sequencing could provide valuable information about the nature of mixed infection and help describe the host’s genetics and immune response to the different genotypes of the infecting strains.^26^ The characterization of samples from the same patients obtained at different points in time could identify genetic components or biomarkers that predict response to therapy or progression of the disease–providing novel therapeutic targets.^26^

## Strengthening the health system

There are critical gaps in the evidence base regarding the current state of health services for Chagas disease in the US and an urgent need for implementation science research to design and test potential scalable models of care for this disease. With respect to health services research, there is a need to both map the care continuum for people with Chagas disease and examine its geographic and sociodemographic variation. Furthermore, models of care must be attentive to the potential for stigma and mistrust among populations at greatest risk of this disease, as well as impediments to healthcare access and ways to overcome them. This research will require strong links to the communities affected and ideally, utilise human-centred design to increase effectiveness. Research conducted locally in single communities has already shown that there may be particular challenges to linking patients who screen positive for Chagas disease to services for diagnostic confirmation and clinical evaluation.^21^ The COVID-19 pandemic further highlighted the vulnerability of the population at risk of Chagas disease to public healthcare service interruptions. Finally, both research and advocacy efforts should emphasise the need for a national awareness strategy for both healthcare providers and the public as well as the public health surveillance systems to monitor progress in strengthening systems of care. Inclusion of Chagas disease screening and case management into relevant clinical guidelines of different American medical associations could help increase awareness.

## Local, state and national programs for neglected tropical diseases

Those who are at risk for or living with Chagas disease in the US are typically Latin American persons who have immigrated from endemic regions and are often under-resourced and marginalised.^6^ Access to appropriate healthcare is often limited to where an individual is living and depends on local, state, and federal resources. For example, the states of Florida and Texas are areas of the US where the burden of Chagas disease is likely high, but they are among the 10 states that have not expanded Medicaid healthcare services and have uninsured rates between 12% and 18% respectively.^27^^,^^28^ Massachusetts, on the other hand, passed legislation in 2006 which allows nearly every resident living within this state to access free or highly subsidized health care insurance. In 2021, only 2.4% of Massachusetts residents were uninsured compared to a national average of 9.2%.^29^ Furthermore, people who acquire autochthonous *T. cruzi* infection in the US commonly live in rural settings; another marginalised population which commonly lacks access to healthcare. There are currently no specific state or federal programs in the US that fund or support those living with Chagas disease. Such programs should include appropriate access to screening, diagnostic testing, and treatment. Chagas disease is not commonly reported to state health departments, being reportable only in a handful of states (Texas, Arizona, Arkansas, Tennessee, Mississippi, Louisiana, and Utah) and Los Angeles county,^30^ greatly hampering our understanding of the actual national burden of this disease. Internationally, programs with strong coordination from national and local health authorities in different epidemiological contexts including Bolivia, Argentina, and Colombia, have successfully increased access to diagnosis and treatment, while Brazil recently made chronic Chagas disease nationally reportable.^31^

People living with Chagas disease in the US need more robust state and federal support to help manage this potentially fatal chronic infection. Two examples of federally funded programs for chronic infectious diseases that can affect vulnerable populations include the Health Resources & Services Administration (HRSA) Ryan White HIV/AIDS Program and the HRSA National Hansen’s Disease (Leprosy) Program.^32^^,^^33^ Each of these programs supports comprehensive care for people living with either HIV or Hansen’s disease. Both are federally funded and allow for people living in the continental US, Puerto Rico, or other US territories to receive comprehensive medical care for both diseases. For example, 14 Hansen’s Disease Ambulatory Care Clinics are located throughout the United States. The main clinical site in Baton Rouge, Louisiana, also provides advanced testing, consultation, and research opportunities for those with Hansen’s disease. Interestingly, with such a robust program for the diagnosis and treatment of Hansen’s disease, only 159 new cases were reported in the US in 2020.^33^ In contrast, the estimated prevalence of Chagas disease in Latin American born adults in the US (i.e., 17,377,709) is estimated to be 1.64.^3^ A similar national program for Chagas disease, mirroring services provided by Ryan White HIV/AIDS and National Hansen’s Disease programs, would ultimately change the landscape of Chagas in our country.

The *STOP (Study, Treat, Observe, and Prevent) Neglected Diseases of Poverty Act*, proposed by US Senator Cory Booker on October 23rd, 2019, provided one potential path toward strengthening the role of US institutions in control of Chagas and other neglected diseases. This proposed legislative action would provide federal funding for Neglected Tropical Diseases (NTDs) in the United States. This bill is the first of its kind in the US and contains several pillars, including (1) creating an interagency task force that would provide advice and recommendations to the Secretary of Health and Human Services and Congress, (2) provide resources to states to implement surveillance systems to determine prevalence and funding to federally qualified health centres to prevent, diagnose and treat those affected, (3) require the development and implementation of educational programs to raise awareness of neglected diseases of poverty, (4) and lastly enable research to develop new and affordable diagnostic tools and treatments, and support one or more centres of excellence for neglected diseases of poverty.^34^ Unfortunately, it did not receive a vote at the 116th Congress (2019–2021) but was re-introduced in the 118th Congress (2022–2023) on 02/09/2023 and referred to the Committee on Health, Education, Labor and Pensions at the time of manuscript generation.^35^

In sum, while US institutions have enormous potential to help end the neglect of diseases such as Chagas, key knowledge gaps persist both in North America and beyond. US research institutions have immense expertise for tackling critical challenges such as identification of biomarkers of disease progression or development of improved diagnostic and therapeutic tools, and many US investigators are already leading the way. The health of vulnerable populations such as those living with Chagas disease is of our collective interest and responsibility. A cohesive, comprehensive, and centralised national program with a research and clinical agenda is in par with meeting the goals set by the WHO’s 2030 neglected tropical diseases road map and long overdue.

## Contributors

Conceptualization and Design: CF and NB. Original draft writing: NA, NB, CF, AFH, JMG. Critical review and editing of the manuscript: NA, NB, CF, AFH, JMG.

## Declaration of interests

The authors report no conflicts of interest.

## Appendix ASupplementary data

Appendix 1

Chagas Research Group list
