# Supplementary material for: Chagas disease in the United States: a call for increased investment and collaborative research
Source: Lancet Reg Health Am. 2024 May 17;34:100768. doi: 10.1016/j.lana.2024.100768 (PMC11127192; doi:10.1016/j.lana.2024.100768)
Supplement: Appendix 1 [file mmc1.docx]

**Appendix 1. The US Chagas Research Group**

Daniel Bourque, Section of Infectious Diseases, Boston University Chobanian & Avedisian School of Medicine

Natalie M. Bowman, University of North Carolina School of Medicine

Malwina Carrion, Boston University and Drugs for Neglected Diseases initiative

Christina Coyle, Albert Einstein College of Medicine

Madolyn Dauphinais, Boston Medical Center

Kelly DeToy, Johns Hopkins University Bloomberg School of Public Health

Robert Gilman, Johns Hopkins University Bloomberg School of Public Health

Davidson H. Hamer, Boston University School of Public Health and Boston University Chobanian & Avedisian School of Medicine

Jesica Herick, University of Chicago School of Medicine

Salvador Hernandez, San Francisco Medical Center

Claudia Herrera, Tulane University School of Public Health and Tropical Medicine

Rachel Marcus, Latin American Society of Chagas and Medstar Union Memorial Hospital

Sheba Meymandi, Olive View-UCLA Medical Center

Melissa Nolan, University of South Carolina Arnold School of Public Health

Katherine Reifler, Boston Medical Center and Boston University Boston University Chobanian & Avedisian School of Medicine

Adrienne Showler, National Institute of Allergy and Infectious Diseases and MedStar Georgetown University Hospital

Paula Stigler Granados, San Diego State University School of Public Health

Anshule Takyar, Johns Hopkins University Bloomberg School of Public Health

Kawsar Talaat, Johns Hopkins University Bloomberg School of Public Health

Shilah Waters, Johns Hopkins University Bloomberg School of Public Health

Alyse Wheelock, Boston Medical Center
