# Supplementary material for: Chagas disease in the United States: a call for increased investment and collaborative research
Source: Lancet Reg Health Am. 2024 May 17;34:100768. doi: 10.1016/j.lana.2024.100768 (PMC11127192; doi:10.1016/j.lana.2024.100768)
Supplement: Chagas Research Group list [file mmc2.docx]

**Chagas Research Group Members**

| **First name** | **Surname** |
| --- | --- |
| Daniel | Bourque |
| Natalie M. | Bowman |
| Malwina | Carrion |
| Christina | Coyle |
| Madolyn | Dauphinais |
| Kelly | DeToy |
| Robert | Gilman |
| Davidson | H. Hamer |
| Jesica | Herick |
| Salvador | Hernandez |
| Claudia | Herrera |
| Rachel | Marcus |
| Sheba | Meymandi |
| Melissa | Nolan |
| Katherine | Reifler |
| Adrienne | Showler |
| Paula | Stigler Granados |
| Anshule | Takyar |
| Kawsar | Talaat |
| Shilah | Waters |
| Alyse | Wheelock |
